# Posterior-Only Vertebral Column Resection for Neglected Post-traumatic Thoracolumbar Fracture-Dislocation With Neurological Recovery: A Case Report

**DOI:** 10.7759/cureus.104815

**Published:** 2026-03-07

**Authors:** Reyaz Ahmed, Abhishek Raina, Sumit Mishra, Umesh Bahagotia, Anirudh Dwajan

**Affiliations:** 1 Orthopaedics, Sher-i-Kashmir Institute of Medical Sciences (SKIMS) Medical College and Hospital, Srinagar, IND; 2 Orthopaedics, All India Institute of Medical Sciences, Bilaspur, Bilaspur, IND

**Keywords:** adult spinal deformity, neglected spinal injury, neurological recovery, post-traumatic kyphosis, post-traumatic spinal deformity, reconstructive surgical procedures, sagittal balance, spinal fusion, thoracolumbar fracture-dislocation, vertebral column resection

## Abstract

Severe neglected post-traumatic thoracolumbar fracture-dislocation may result in rigid kyphotic deformity, sagittal imbalance, chronic pain, and delayed neurological compromise. We report the case of a middle-aged patient who presented with symptomatic rigid thoracolumbar kyphosis three years after an inadequately treated vertebral fracture. Radiological evaluation demonstrated a fixed angular deformity with translational instability and significant sagittal imbalance. The patient underwent single-stage, posterior-only, vertebral column resection with long-segment pedicle screw fixation and circumferential anterior column reconstruction. Adequate deformity correction and restoration of sagittal alignment were achieved without intraoperative neurological deterioration. Postoperatively, the patient demonstrated significant improvement in pain, posture, and functional mobility, with neurological recovery from American Spinal Injury Association (ASIA) Impairment Scale grade D preoperatively to ASIA grade E at final follow-up. This case highlights posterior-only vertebral column resection as an effective salvage strategy in severe neglected post-traumatic thoracolumbar deformity when limited osteotomies are unlikely to achieve adequate decompression or alignment restoration.

## Introduction

Post-traumatic thoracolumbar kyphosis is a recognized late sequela of inadequately treated spinal fractures and may lead to persistent pain, progressive deformity, sagittal imbalance, and neurological impairment [1,2]. When diagnosis or definitive stabilization is delayed, gradual vertebral collapse and translational deformity may occur, particularly at the thoracolumbar junction, which represents a biomechanical transition zone prone to instability [3]. While mild deformities may respond to conservative treatment or limited corrective osteotomies, rigid kyphosis associated with fixed translation and spinal canal compromise presents a distinct surgical challenge [4,5]. In such situations, indirect decompression techniques are often inadequate. Posterior vertebral column resection allows single-stage three-column correction with direct neural decompression through a posterior-only approach and is reserved for selected cases of severe post-traumatic deformity where limited osteotomies are unlikely to achieve adequate correction [6-8].

## Case presentation

A 26-year-old woman presented to our tertiary spine unit with persistent low back pain, progressive deformity at the dorsolumbar junction, and right-sided lower limb weakness of three years’ duration. The symptoms followed a fall from a height of approximately 12 feet, after which she developed acute back pain with bilateral lower limb weakness and inability to ambulate independently. During the first year following injury, partial neurological recovery occurred in the left lower limb, allowing assisted ambulation. However, right lower limb weakness persisted without meaningful improvement. Over time, the patient developed progressively worsening mechanical back pain and a gradual increase in kyphotic deformity, resulting in compensatory postural imbalance and functional limitation.

Over subsequent months, gradual neurological recovery occurred in the left lower limb, allowing partial restoration of motor function. In contrast, right-sided weakness persisted, and the patient developed progressively worsening mechanical low back pain. The pain was aggravated by upright posture, prolonged sitting, and physical activity, and was partially relieved by rest and recumbency. There was no history of bowel or bladder dysfunction, sensory disturbance, or constitutional symptoms. Despite intermittent physiotherapy over a three-year period, the patient continued to experience persistent right lower limb weakness and progressively worsening mechanical back pain. Over the six months preceding presentation, she reported a marked increase in pain severity, progressive spinal deformity, and increasing difficulty with prolonged standing and ambulation, which significantly impaired her daily activities. These worsening symptoms, along with visible progression of deformity and persistent neurological deficit, prompted referral to our tertiary spine center for definitive surgical evaluation and management. Preoperatively, the patient reported severe mechanical back pain with a Visual Analog Scale (VAS) score of 8/10, which significantly impaired daily activities.

Clinical examination revealed a visible gibbus deformity at the dorsolumbar junction with loss of lumbar lordosis and mild coronal imbalance. Neurological examination demonstrated motor weakness in the right lower limb. Muscle strength was graded using the Medical Research Council (MRC) scale as follows: hip flexion 4/5, knee extension 3/5, ankle dorsiflexion 3/5, great toe extension 3/5, and ankle plantarflexion 4/5. Motor strength in the left lower limb was normal (MRC grade 5/5 across all muscle groups). Sensory examination was intact bilaterally. Deep tendon reflexes were diminished at the right knee and ankle. There was no bowel or bladder dysfunction. The patient was classified as American Spinal Injury Association (ASIA) Impairment Scale grade D. Higher mental functions were intact, with no signs of upper motor neuron involvement. 

**Figure 1:**
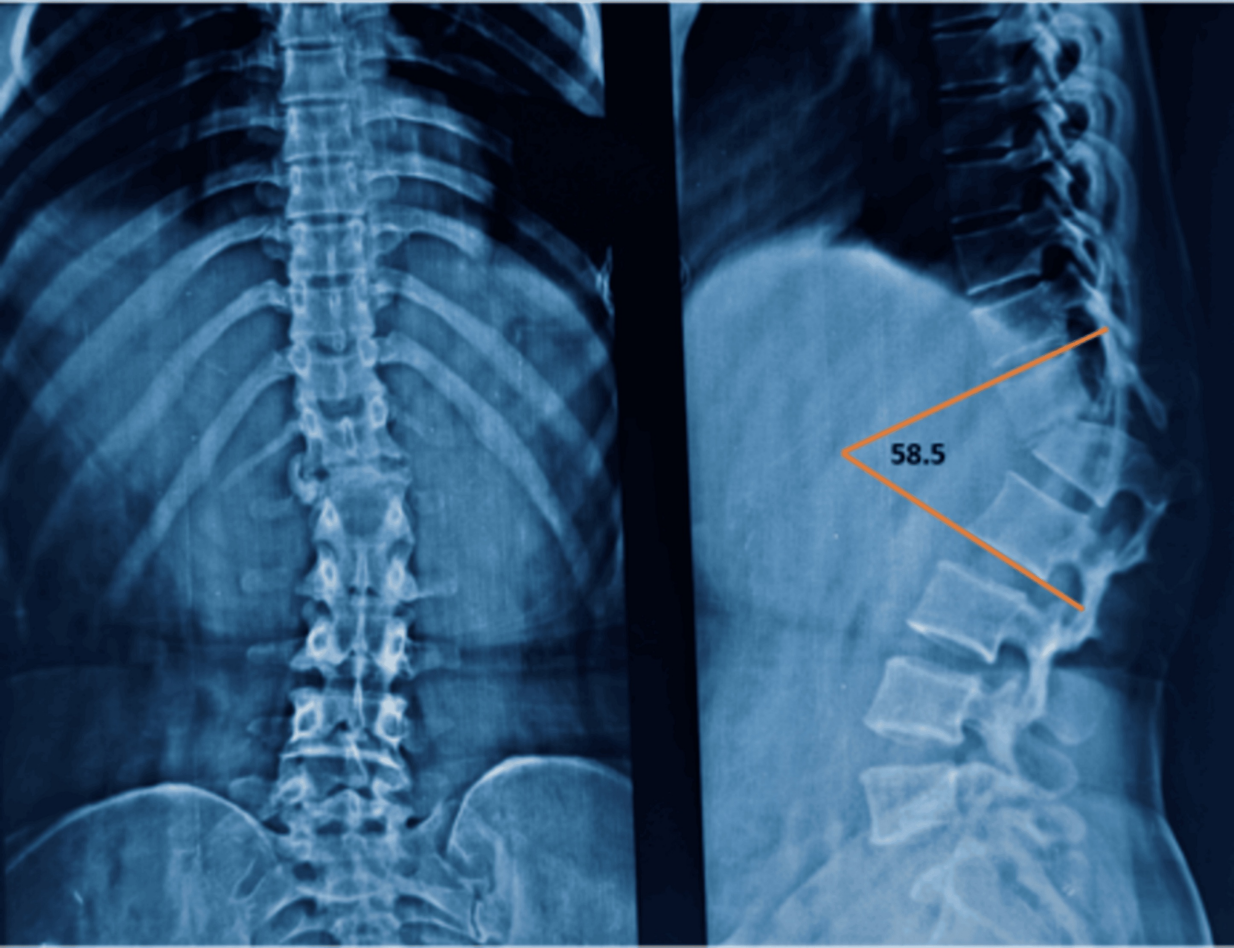
Preoperative plain radiographs of the thoracolumbar spine. (A) Anteroposterior view demonstrating coronal alignment. (B) Lateral view showing severe post-traumatic kyphosis at D12–L1 with vertebral body collapse and fixed translational deformity. The focal kyphotic angle measured 58.5° using the Cobb method.

Plain radiographs demonstrated severe kyphotic deformity with vertebral body collapse and translational displacement at the D12-L1 level (Figure 1). The magnitude of kyphotic deformity was measured using the Cobb method on standing lateral radiographs, defined as the angle between the superior endplate of D12 and the inferior endplate of L1. These measurements were obtained preoperatively, postoperatively, and at final follow-up to objectively assess deformity severity and correction. The preoperative focal kyphosis measured 58.5° using the Cobb method (superior endplate of D12 to inferior endplate of L1). Immediate postoperative radiographs demonstrated correction to 14.9°, representing an angular correction of 43.6° (74.5%). Alignment was maintained at final follow-up without evidence of loss of correction or implant failure.

**Figure 2:**
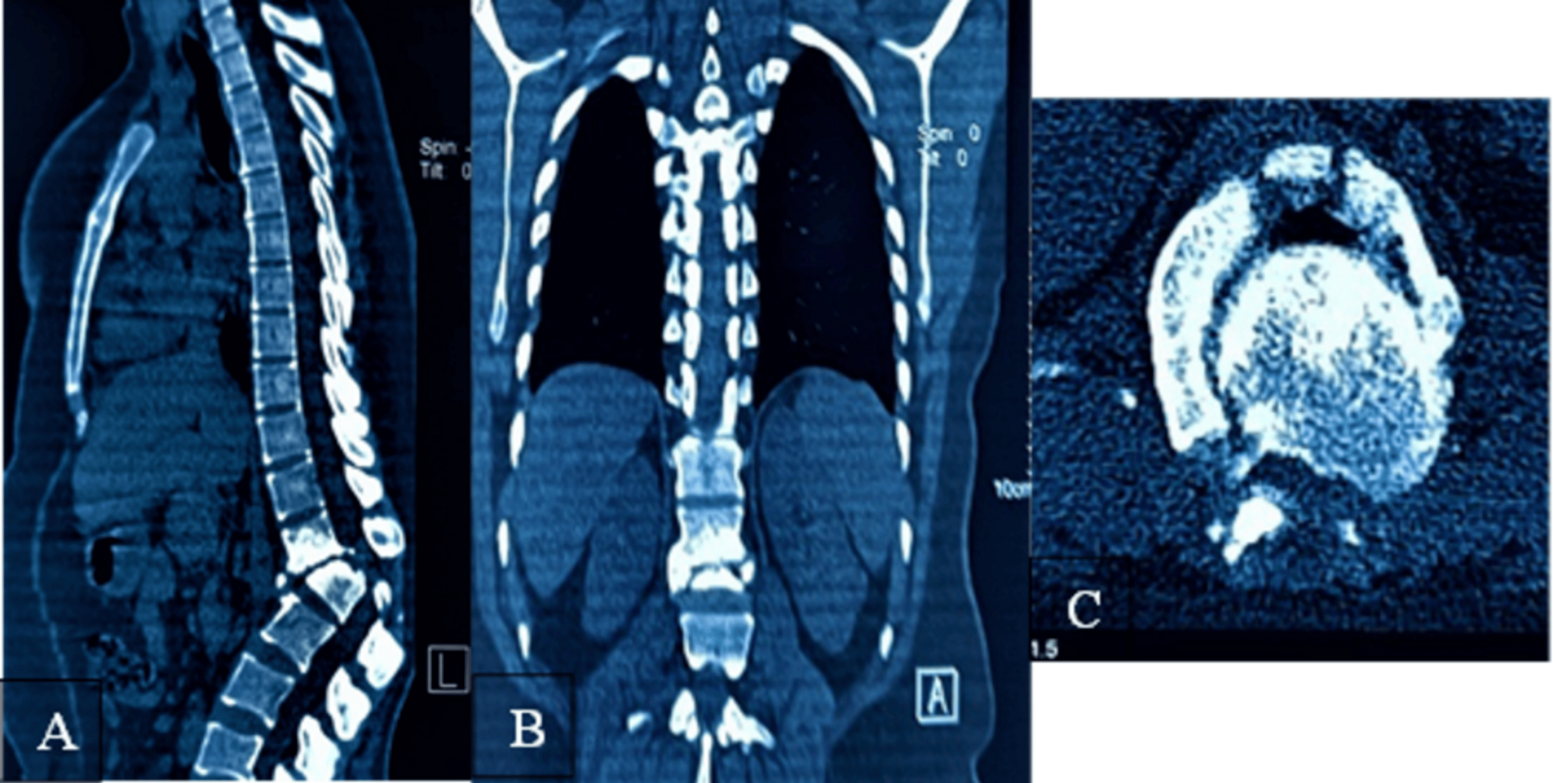
Preoperative computed tomography of the thoracolumbar spine. (A) Sagittal view showing L1 vertebral collapse and kyphotic deformity. (B) Coronal view demonstrating disrupted alignment. (C) Axial view showing severe canal compromise.

Computed tomography demonstrated comminution of the L1 vertebral body and a fixed translational deformity consistent with rigid post-traumatic fracture-dislocation (Figure 2).

**Figure 3:**
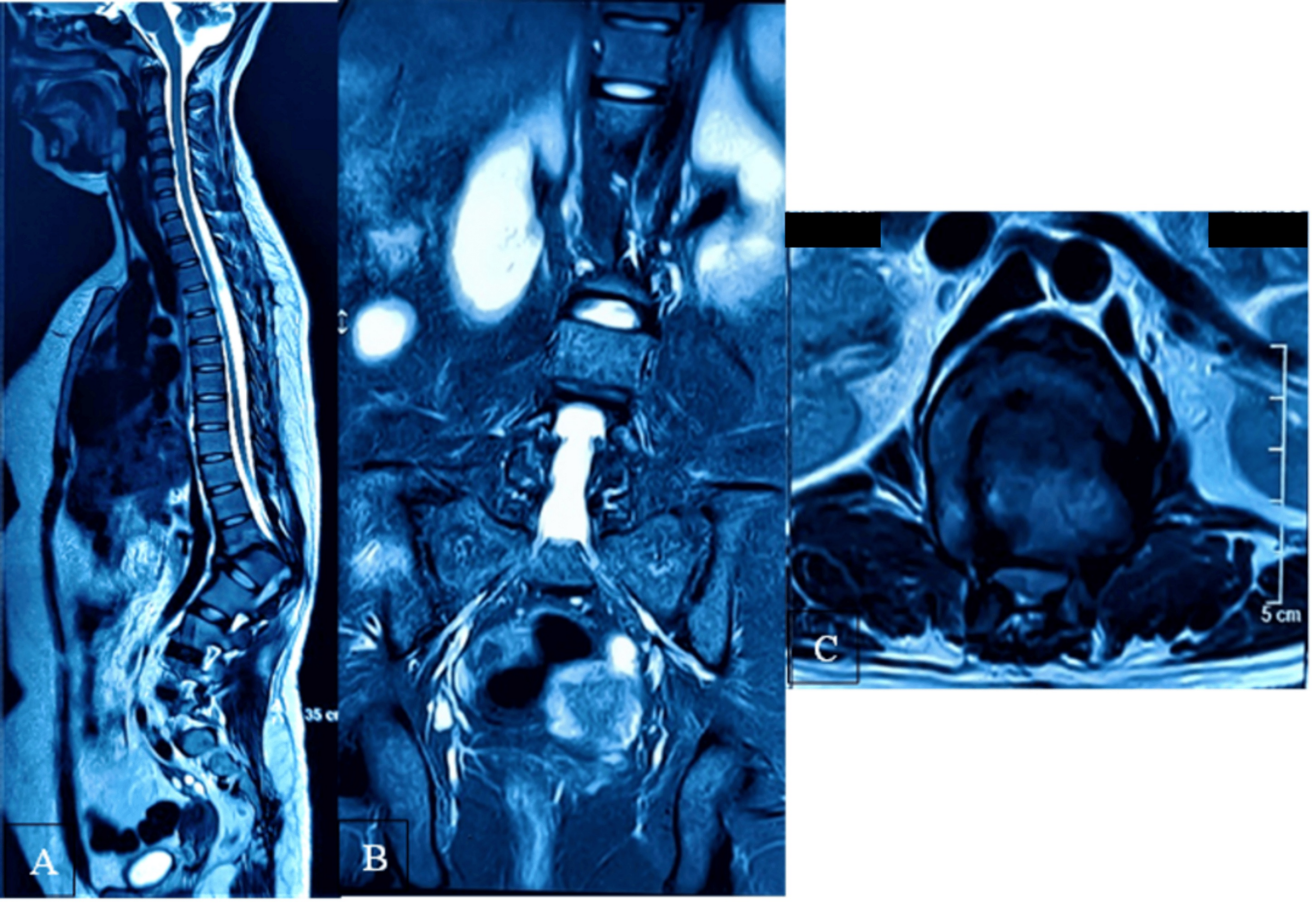
Preoperative magnetic resonance imaging of the thoracolumbar spine. (A) Sagittal view showing kyphotic deformity and canal narrowing at D12–L1. (B) Coronal view demonstrating loss of alignment. (C) Axial view showing critical thecal sac compression.

Magnetic resonance imaging demonstrated a burst fracture of the L1 vertebral body with marked anterior wedging and grade four traumatic spondylolisthesis of D12 over L1, resulting in critical canal compromise with a minimum canal diameter of approximately 4 mm. Disruption of the posterior ligamentous complex confirmed chronic instability (Figure 3).

Given the combination of severe kyphosis, fixed translational deformity, anterior column deficiency, and circumferential canal compromise, a single-stage, posterior-only, vertebral column resection was performed under continuous intraoperative neuromonitoring using somatosensory evoked potentials (SSEP) and motor evoked potentials (MEP). Long-segment posterior fixation from D10 to L2 was established using ten polyaxial 6.5-mm pedicle screws, and temporary rods were applied to maintain stability during resection. Controlled vertebral column resection at D12-L1 allowed complete circumferential decompression of the spinal cord and nerve roots, creating a safe corridor for deformity correction and anterior column reconstruction. Gradual deformity correction and controlled posterior column shortening were performed using temporary rods, which reduced tension on the neural elements and facilitated safe reconstruction. The dura and nerve roots were carefully mobilized and protected using malleable dural and nerve root retractors under direct visualization. The anterior column defect was prepared using angled curettes and pituitary rongeurs.

**Figure 4:**
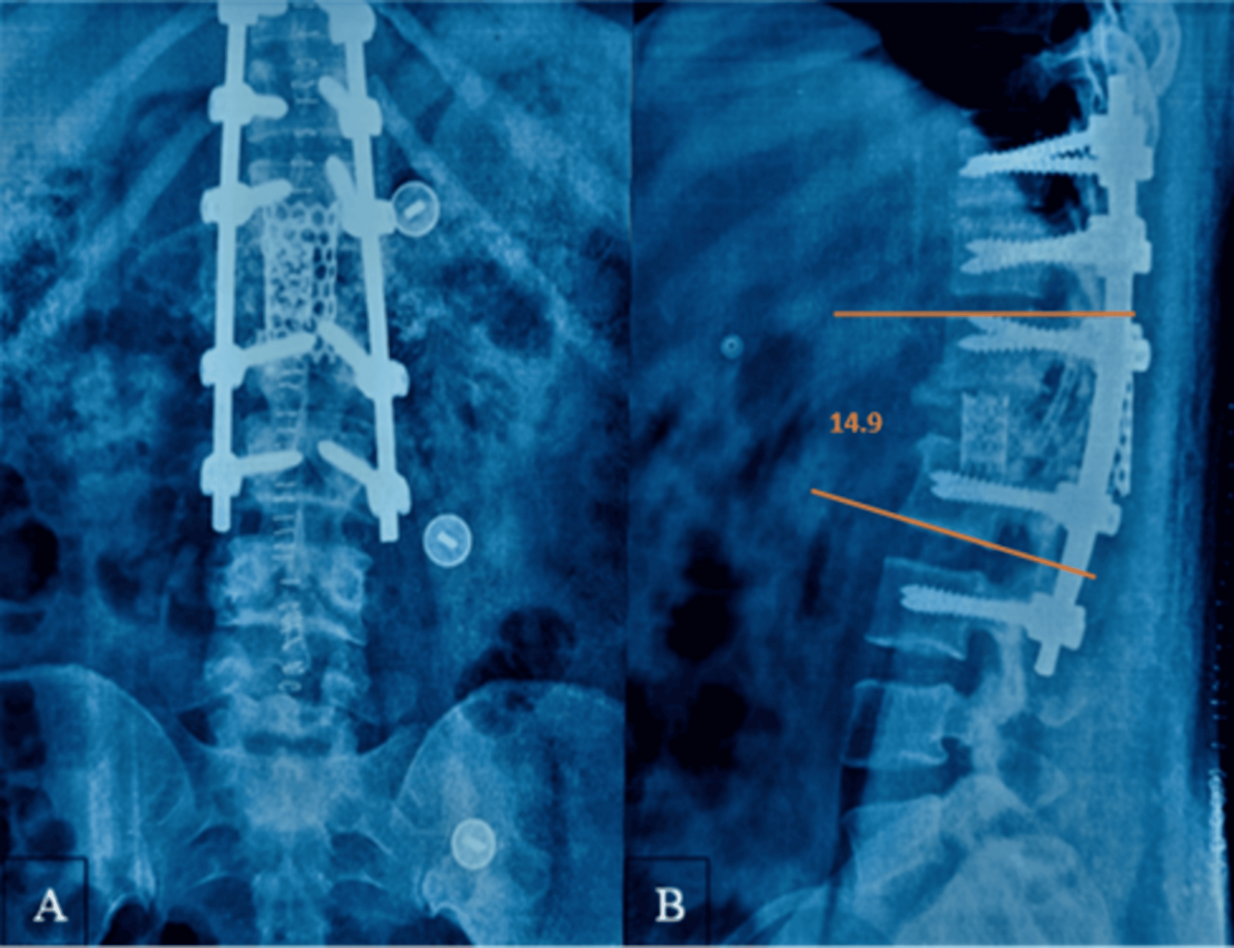
Immediate postoperative plain radiographs. (A) Anteroposterior view demonstrating long-segment posterior instrumentation from D10 to L2. (B) Lateral view showing correction of focal kyphosis from 58.5° preoperatively to 14.9° postoperatively following posterior vertebral column resection and anterior column reconstruction with titanium mesh cages.

A cylindrical titanium mesh cage measuring 20 mm in diameter and 26 mm in length, packed with autologous bone graft harvested during vertebral resection, was inserted obliquely through the posterior corridor into the anterior column space using specialized cage holders under fluoroscopic guidance. Care was taken to avoid excessive neural retraction, and neuromonitoring signals remained stable throughout cage insertion. A second titanium mesh cage measuring 18 mm in diameter and 22 mm in length was then positioned posteriorly within the defect to provide additional structural support and ensure circumferential anterior column reconstruction. Final stabilization was achieved using contoured cobalt-chromium rods, and satisfactory deformity correction and implant positioning were confirmed fluoroscopically (Figure 4). Continuous neuromonitoring demonstrated stable signals throughout vertebral column resection, deformity correction, and cage placement, confirming the absence of intraoperative neurological compromise. The total operative time was 3.5 hours, and the estimated intraoperative blood loss was 800 mL. The patient remained hemodynamically stable throughout the procedure, and no intraoperative complications were encountered.

Postoperatively, the patient was managed in the intensive care unit and gradually mobilized using a custom-made thoracolumbar brace. A superficial incisional surgical site infection developed during the early postoperative period, presenting with localized erythema and serous discharge. Wound cultures were negative. The patient was treated empirically with intravenous cefazolin followed by oral cephalexin for a total duration of 15 days, resulting in complete resolution without implant involvement or need for surgical intervention. Gradual neurological improvement in right lower limb motor strength was observed. Sensory function and bowel and bladder control remained preserved throughout recovery.

**Figure 5:**
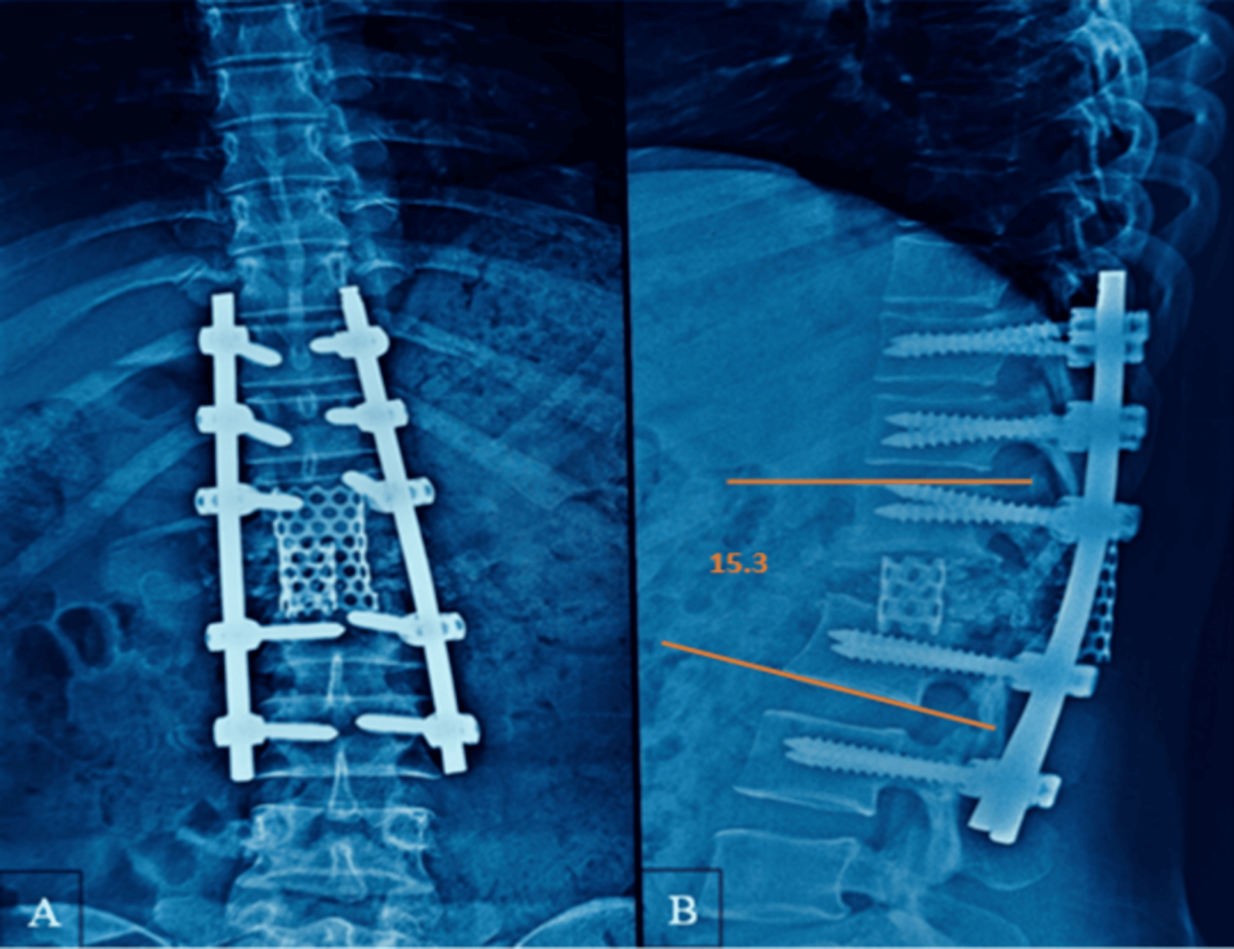
Figure 5. Two-year follow-up plain radiographs. (A) Anteroposterior view demonstrating stable posterior instrumentation from D10 to L2 without evidence of implant loosening or failure. (B) Lateral view showing maintained correction of focal kyphosis (14.9°) with preserved sagittal alignment and radiographic evidence of solid fusion across the reconstructed segment.

At the two-year follow-up, the patient was independently ambulatory and reported marked relief from back pain. Neurological function improved from preoperative ASIA Impairment Scale grade D to ASIA grade E at final follow-up, confirming complete neurological recovery. Radiographs demonstrated maintained deformity correction, stable instrumentation, and solid fusion without loss of alignment. Neurological improvement was sustained at final follow-up (Figure 5). The patient reported substantial pain improvement, with VAS score improving from 8/10 preoperatively to 3/10, reflecting excellent clinical recovery.

## Discussion

Neglected thoracolumbar trauma should not be considered merely as a healed fracture with residual deformity. Delay in definitive stabilization may permit progressive vertebral collapse, translational deformity, and chronic neural compression, particularly at the thoracolumbar junction, where biomechanical stresses are greatest [1,2]. Over time, these changes may convert a potentially manageable injury into a rigid deformity with limited corrective options [3].

Posterior column osteotomies and pedicle subtraction osteotomy remain well-established techniques for correction of post-traumatic thoracolumbar kyphosis in adults [4,5]. These procedures are effective when deformity is predominantly angular and translational instability is minimal. However, their corrective capacity is limited when translation is fixed, the anterior column is deficient, or the spinal canal is critically compromised [7,9].

In the present case, the coexistence of severe kyphosis, fixed translational deformity, and circumferential canal compromise precluded the use of limited osteotomies. Posterior vertebral column resection enabled controlled three-dimensional (3D) deformity correction while allowing direct visualization and decompression of the neural elements, which was central to achieving neurological recovery [8,10]. Vertebral column resection is more commonly reported in congenital, pediatric, and post-infective spinal deformities, whereas its application in adult post-traumatic kyphosis remains relatively uncommon [10]. Trauma-related cases constitute a small proportion of published series, reflecting both the rarity of appropriate indications and the technical complexity of the procedure [8].

A distinctive and clinically important aspect of this case lies in the management of a long-standing neglected thoracolumbar fracture-dislocation with fixed translational deformity using a posterior-only, single-stage vertebral column resection combined with circumferential anterior column reconstruction using dual titanium mesh cages. Reports describing posterior-only circumferential reconstruction with dual structural cage placement in chronic post-traumatic translational deformities remain limited. This approach provided robust anterior column support while avoiding the additional morbidity associated with staged anterior-posterior procedures [9,10].

Posterior vertebral column resection enables circumferential decompression and anterior column reconstruction through a posterior-only approach by creating a controlled anterior defect and permitting controlled posterior column shortening. This reduces tension on neural structures and allows safe cage placement under direct visualization. Careful neural protection using specialized retractors, gradual deformity correction, and continuous intraoperative neuromonitoring are critical to minimizing neurological risk during reconstruction.

Although single large-footprint expandable cages are increasingly used for anterior column reconstruction, the use of dual static titanium mesh cages offered specific biomechanical and technical advantages in this patient. Severe translational deformity and asymmetric vertebral collapse created an irregular anterior column defect, making safe insertion and deployment of a single expandable cage technically challenging through a constrained posterior-only corridor. The use of two smaller-profile cages allowed controlled sequential insertion with minimal neural manipulation. Furthermore, dual cage placement facilitated more uniform axial load distribution, reducing the risk of subsidence and improving construct stability in the setting of chronic deformity and altered biomechanics. Titanium mesh cages packed with autologous bone graft also provide favorable osteoconductive properties, promoting biological fusion. This strategy enabled safe circumferential reconstruction while achieving durable deformity correction and minimizing neurological risk.

Surgical correction of post-traumatic thoracolumbar deformity is indicated in patients with progressive deformity, persistent mechanical pain, neurological deficit, or significant sagittal imbalance. In neglected injuries, progressive vertebral collapse and fixed translational deformity may result in chronic neural compression and functional impairment, necessitating surgical intervention. Vertebral column resection is particularly indicated in rigid deformities with translational instability or severe canal compromise, where limited osteotomies are unlikely to achieve adequate decompression or alignment restoration.

An important observation in this case was the significant neurological recovery despite delayed presentation, with improvement from ASIA Impairment Scale grade D to grade E. This highlights the potential reversibility of neurological deficits in selected patients with chronic deformity when adequate neural decompression and biomechanical stabilization are achieved.

This report has inherent limitations as a retrospective single-case observation, which limits generalizability. Clinical outcomes may vary depending on deformity severity, duration, and individual patient factors. Larger studies with longer follow-up are needed to further evaluate the safety, biomechanical effectiveness, and reproducibility of posterior-only vertebral column resection with dual cage reconstruction in neglected thoracolumbar fracture-dislocation.

## Conclusions

Posterior vertebral column resection provided effective surgical management of this severe neglected post-traumatic thoracolumbar fracture-dislocation with rigid deformity and translational instability. Posterior-only circumferential reconstruction using dual titanium mesh cages restored anterior column support and enabled objective deformity correction, as demonstrated by improvement in segmental kyphosis measured by Cobb angle and restoration of global sagittal alignment assessed by sagittal vertical axis. Meaningful neurological recovery from ASIA Impairment Scale grade D to grade E was also observed. This case highlights that carefully planned posterior vertebral column resection with circumferential reconstruction can achieve neural decompression, deformity correction, and functional recovery even in delayed presentations. Recognition of neglected spinal injuries and individualized surgical planning remain essential for optimizing outcomes in such complex deformities.

## References

[1] Khatri K, Farooque K, Sharma V, Gupta B, Gamanagatti S (2016). Neglected thoraco lumbar traumatic spine injuries. Asian Spine J.

[2] Kanna RM, Khurjekar K (2018). Thoracolumbar trauma with delayed presentation. Ind Spine J.

[3] Yaman O, Zileli M, Şentürk S, Paksoy K, Sharif S (2021). Kyphosis after thoracolumbar spine fractures: WFNS Spine Committee recommendations. Neurospine.

[4] Buchowski JM, Kuhns CA, Bridwell KH, Lenke LG (2008). Surgical management of posttraumatic thoracolumbar kyphosis. Spine J.

[5] Chou D, Wang VY, Storm PB (2010). Pedicle subtraction osteotomies for the correction of post-traumatic thoracolumbar kyphosis. J Clin Neurosci.

[6] Munting E (2010). Surgical treatment of post-traumatic kyphosis in the thoracolumbar spine: indications and technical aspects. Eur Spine J.

[7] Lenke LG, Sides BA, Koester LA, Hensley M, Blanke KM (2010). Vertebral column resection for the treatment of severe spinal deformity. Clin Orthop Relat Res.

[8] El Naggar A, Elgawhary S, ElHewala T (2018). Posterior vertebral column resection in management of severe post-traumatic thoracolumbar kyphosis. Adv Spine J.

[9] Hamzaoglu A, Elsadig M, Karadereler S (2022). Single-stage posterior vertebral column resection with circumferential reconstruction for thoracic/thoracolumbar burst fractures with or without neurological deficit: clinical neurological and radiological outcomes. Global Spine J.

[10] Saifi C, Laratta JL, Petridis P, Shillingford JN, Lehman RA, Lenke LG (2017). Vertebral column resection for rigid spinal deformity. Global Spine J.

